# Evaluating the Picterus Jaundice Pro App for Neonatal Jaundice Screening by Parents: Prospective Mixed Methods Study

**DOI:** 10.2196/83077

**Published:** 2026-07-27

**Authors:** Frouke J Terpstra, Ariela Hazan, Elkana Kohn, Matitiahu Berkovitch, Tjalling W de Vries, Jasper V Been, Peter H Dijk, Christian V Hulzebos

**Affiliations:** 1 Department of Neonatology Beatrix Children’s Hospital University Medical Center Groningen Groningen The Netherlands; 2 Clinical Pharmacology and Toxicology Gray School of Medicine, Tel Aviv University Shamir Medical Center (Assaf Harofeh) Zerifin Israel; 3 Clinical Pharmacology and Toxicology, Pediatric Division Gray School of Medicine, Tel Aviv University Shamir Medical Center (Assaf Harofeh) Zerifin Israel; 4 Department of Pediatrics Frisius Medical Center Leeuwarden The Netherlands; 5 Division of Neonatology, Department of Neonatal and Paediatric Intensive Care Erasmus MC Sophia Children’s Hospital, University Medical Centre Rotterdam Rotterdam The Netherlands; 6 Department of Obstetrics and Gynaecology Erasmus MC Sophia Children’s Hospital, University Medical Centre Rotterdam Rotterdam The Netherlands

**Keywords:** bilirubin screening, clinical decision support, digital health, hyperbilirubinemia, jaundice detection, mobile health, neonatal jaundice

## Abstract

**Background:**

Neonatal jaundice is a very common condition in the first week of life requiring timely recognition of the small subset of neonates at risk of developing severe neonatal hyperbilirubinemia (SNH). Total serum bilirubin, as quantified through blood testing, remains the gold standard for diagnosis, whereas screening based on visual inspection has been proven unreliable in identifying which jaundiced neonates require total serum bilirubin. Picterus Jaundice Pro (JP) is a mobile health app developed for use by health care professionals and parents to screen for imminent SNH. However, parental user experiences with Picterus JP remain largely unexplored.

**Objective:**

This study aimed to evaluate the usability and user-friendliness of Picterus JP when used and evaluated by parents.

**Methods:**

Between August 2023 and January 2025, we conducted a prospective mixed methods study at 3 maternity care settings: the maternity wards of 2 hospitals in the Netherlands and 1 hospital in Israel. Parents were invited to participate if their infant was near term born (gestational age of ≥35 weeks) and at least 12 hours old but still in the first week of life. Parents used Picterus JP once according to the manufacturer’s instructions. Immediately afterward, usability was evaluated using the validated mHealth App Usability Questionnaire, an instrument measured on a 7-point Likert scale ranging from 1 (“strongly disagree”) to 7 (“strongly agree”), and open-ended questions. A subset of parents and nurses participated in semistructured interviews and focus groups.

**Results:**

In total, 123 parents completed the questionnaire. The median mHealth App Usability Questionnaire score was 6 (IQR 5-7) out of 7. A total of 87.7% (100/114) of the parents indicated that they would use Picterus JP at home. Parents reported that they found the app easy, quick, and convenient, although 55.8% (67/120) reported a failure on the first try. Some parents struggled to find or interpret the instructions, but most of those who tried again (79/105, 75.2%) obtained a bilirubin estimate. Additionally, 12 nurses from 2 participating maternity wards provided their observations on parents’ use of Picterus JP in 2 focus groups. Thematic analysis of all combined data identified 3 themes that were considered important for the usability of Picterus JP: intention to use Picterus JP, infant well-being, and instruction and guidance.

**Conclusions:**

Parents rated Picterus JP highly in terms of usability despite encountering difficulties during initial use. Technical improvements and more proactive guidance seem important to support successful parental use. Nonetheless, both parents and nurses were positive about the potential of Picterus JP for home screening for SNH.

## Introduction

Neonatal jaundice, a yellowish discoloration of the skin and sclerae, occurs in the first days of life due to excessive levels of total serum bilirubin (TSB) [1]. Neonatal jaundice affects over 80% of neonates and is generally self-limiting [2]. However, in 4% to 9% of near-term neonates, treatment is necessary to prevent neonatal jaundice from progressing to severe neonatal hyperbilirubinemia (SNH) [3,4]. Undiagnosed and untreated SNH can lead to irreversible brain damage (ie, kernicterus). Kernicterus is a preventable condition that leads to sensorineural hearing loss, vertical gaze paralysis, dystonia, or more subtle forms of kernicterus spectrum disorders [5]. Therefore, within the large group of jaundiced infants, it is paramount to detect those who develop potentially dangerous levels of TSB.

The gold standard for diagnosing hyperbilirubinemia is quantification of TSB levels through blood sampling [6]. Screening via transcutaneous bilirubin (TcB) is a reliable method to facilitate early recognition of SNH, but the costly TcB devices are not accessible to everyone everywhere every day [7]. As such, visual inspection is widely used by health care professionals (HCPs) to assess the severity of jaundice, although this has shown to be unreliable and inaccurate [8,9].

In some countries, a predischarge TSB or TcB screening is performed to ensure appropriate follow-up of neonatal jaundice [4]. However, this often occurs before the physiological peak in TSB levels, which is typically around the fourth day of life. Other strategies encompass the use of risk factors to predict the risk of developing SNH [10-12]. However, HCP home visits are typically scheduled once or a few times during the first week of life but do not occur daily. As a result, jaundiced neonates may continue to develop SNH unnoticed, placing them at risk of kernicterus and the need for intensive treatment once diagnosed [13].

To support timely diagnosis and treatment, several novel devices to screen for SNH have been developed [7,14-18]. Only a subset of these approaches uses smartphone apps, allowing for bilirubin screening using widely available smartphones rather than dedicated devices.

One of these apps is the Picterus Jaundice Pro (JP), a mobile health (mHealth) app developed to estimate bilirubin levels noninvasively. Picterus JP showed high sensitivity (94%) in detecting SNH and has obtained European CE marking [19-21]. Parents increasingly use mHealth tools for monitoring in the neonatal period [22]. However, the usability of existing apps varies widely, and usability influences use in real‑world settings [22,23]. By empowering parents to use the app at home, Picterus JP could facilitate frequent screening for imminent SNH at any time. However, parental experiences with Picterus JP remain largely unexplored.

Therefore, this study aimed to determine the usability and user-friendliness of Picterus JP when used and evaluated by parents.

## Methods

### Study Design

We evaluated the usability and user-friendliness of Picterus JP through a prospective mixed methods study using the validated mHealth App Usability Questionnaire (MAUQ), open-ended questions, interviews, and focus groups.

### Setting and Participants

This study was part of the Jaundice@Home project conducted under the Transforming Health and Care Systems program (project number: 1477) [24]. Participants were recruited from 3 distinct real-world newborn care settings: the maternity wards of 2 hospitals in the Netherlands (Frisius Medical Center, Leeuwarden, and University Medical Center Groningen [UMCG]) and 1 in Israel (Shamir Medical Center). This study was initially conducted in the Netherlands from August 2023 to December 2023 (period 1) and extended to Israel from October 2024 to January 2025 (period 2). To ensure comparability, this study was also repeated at UMCG in the Netherlands in the second period.

Parents and/or caregivers were invited to participate if their neonates were healthy, with a gestational age of 35 or more weeks and between the age of 12 hours and 7 days. At least one parent was required to have sufficient proficiency in Dutch (in the Netherlands) or Arabic or Hebrew (in Israel).

### Intervention

Parents were asked to use Picterus JP at least once during their hospital stay. Immediately afterward, parents completed a paper questionnaire about their experiences, which was collected within 24 hours. The app could only be used by parents during their hospital stay for practical and safety considerations. All participating neonates received standard care to ensure safe practice. This included visual inspection for signs of jaundice and TcB and/or TSB measurements as deemed necessary by the attending physicians or midwives. The use of the Picterus JP app did not affect standard care.

All parents received a Picterus calibration card, a download link, and the same instructions for using Picterus JP at their convenience. The instructions consisted of a brief verbal explanation; textual instructions (via the patient information letter or app); and pictograms, which were included in the app and printed out [25]. The study team did not provide explicit demonstrations or guidance before and during app use. Nurses received a demonstration of the app at the beginning of the study period. The study team and nurses were encouraged to leave the room while parents used the app but would sometimes remain as silent bystanders. Parents either installed the app on their own smartphones (the Netherlands) or used a Samsung Galaxy A50 smartphone provided by the researchers (Israel).

**Figure 1:**
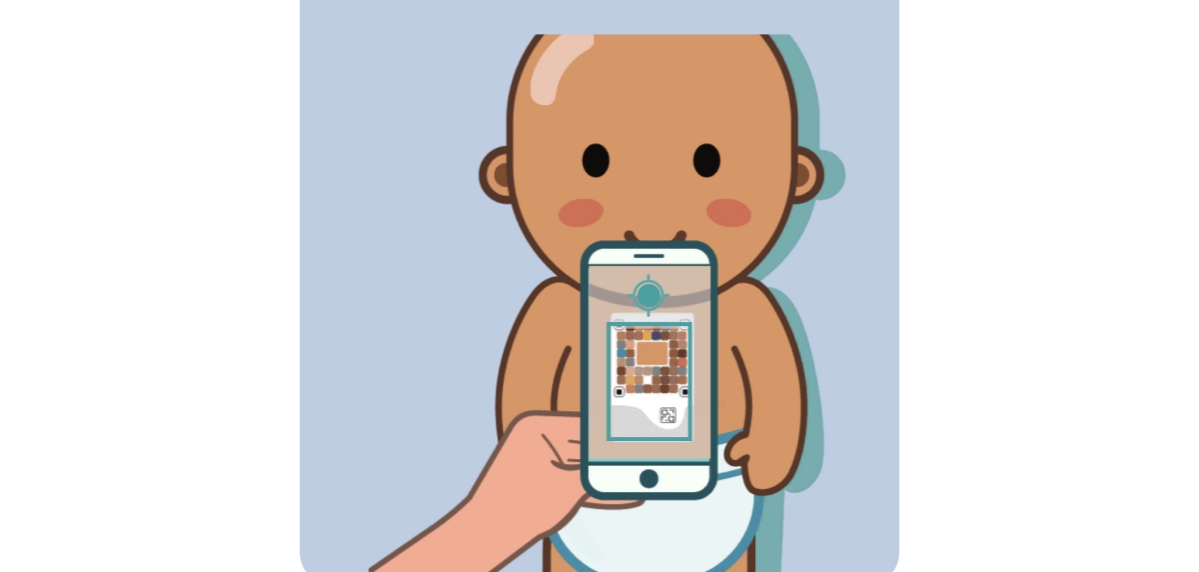
Intended use of Picterus Jaundice Pro. Figure used with permission from Picterus AS.

When starting Picterus JP, users were instructed to place the square, colorful Picterus calibration card on the infants’ bare chest (Figure 1). Picterus JP then guided users on-screen on how to correctly position their smartphone (ie, angle and distance). Once the app detected that the conditions were appropriate (ie, correct placement of the calibration card and positioning of the smartphone and sufficient ambient light), it would then automatically proceed to take 6 photos: 3 with flash and 3 without.

After capturing the images, the app either displayed a bilirubin value (in μmol/L or mg/dL depending on the setting) or issued an error message. If an error occurred, the app either stated that the measurement was unsuccessful or, if possible, provided a reason (eg, insufficient ambient light or shadows rendering the photo unsuitable for analysis). During the photo-taking phase, the app attempted to correct suboptimal conditions by offering simple on-screen instructions to the user. If image quality remained insufficient, Picterus JP stopped the standard 6-image count and either continued attempting automatic image capture or temporarily paused image capture. Image counting resumed only once image quality improved following user adjustment. If adequate image quality could not be achieved, the measurement attempt was eventually aborted and an error message was displayed, when available.

### Data Collection

#### Quantitative Data Collection

The MAUQ was used to evaluate Picterus JP usability. This is a validated instrument specifically designed to evaluate the usability of mHealth apps [26].

The MAUQ consists of 18 statements divided into 3 subscales: ease of use, interface and satisfaction, and usefulness (Table S1 in Multimedia Appendix 1). One statement (number 17) from the original MAUQ version was omitted because it assessed offline functionality, which did not apply. The MAUQ was scored from 1 (“strongly disagree”) to 7 (“strongly agree”) on statements in favor of the device. Statements were translated from English into Arabic, Dutch, and Hebrew and verified via independent back translation.

#### Qualitative Data Collection

All parents were asked to answer 4 open-ended questions about their experiences with the app to elaborate on topics such as their general experience, child comfort, and possible malfunctioning of the app and provide additional comments.

Semistructured interviews and focus groups were conducted using a topic list covering 4 categories: general experience, instruction and guidance, possible app malfunctions, and perceived future use and suggestions for improvement. The topics encompassed 2 to 5 questions that the researcher could use to elicit further responses if participants provided limited information. The topic list can be found in Multimedia Appendix 2.

A subset of 5 participants per center was invited to take part in the semistructured interviews at the midpoint of the study period. Participants were initially approached consecutively at that time point. After the first 2 interviews, additional participants were selected to ensure representation across a broad range of MAUQ scores. The interviews lasted between 5 and 15 minutes and were conducted either in person or over the phone by local members of the study team. The interviews were generally audio recorded and transcribed, whereas phone interviews were documented through detailed notes. Data saturation was considered to have been reached when no new codes or themes emerged from the available data.

Registered nurses in the Netherlands were invited to share their observations in focus groups held at the end of a regular work shift near the end of the study period. The number of focus groups was determined pragmatically rather than through data saturation: one in each center where nurses had observed parents using Picterus JP. Focus groups were led by 2 researchers (FT and TdV) and were recorded and transcribed. Each session lasted between 15 and 25 minutes.

#### Definition of Participant Characteristic Variables

Parental educational level was classified into three categories according to the self-reported highest level attained: (1) basic education (elementary school), (2) intermediate education (high school and vocational training), and (3) advanced education (college or university degree).

Postnatal age was recorded in 24-hour intervals (eg, 24-48 hours and 48-72 hours) based on the time of birth. Daily check-ins were conducted by the study team to confirm whether parents had used the app within the previous 24 hours. Exact age in hours was not documented. Infants younger than 12 hours were not enrolled due to the informed consent reflection period.

Additional demographic and clinical variables collected from infants and parents were neonatal sex, birth weight, gestational age, maternal parity, and country and hospital of recruitment. These variables were collected as per standard clinical documentation.

Limited information on health care providers was collected, including age, years of work experience, and the number of times they observed the use of the app.

### Ethical Considerations

This study was conducted in accordance with the Declaration of Helsinki. In the Netherlands, the study was approved by the Central Ethical Review Committee of UMCG (research register number 10763). This study was classified as falling outside the scope of the Medical Research Involving Human Subjects Act. Local feasibility was confirmed by the Regional Ethics Review Committee at Frisius Medical Center (reference: RTPO 1160). In Israel, the study was approved by Shamir Medical Center Helsinki Committee (approval number 264-23-ASF). Informed consent was obtained from the parents of all participating neonates.

### Data Analysis

#### Sample Size

A convenience sample of 120 participants was targeted across 2 study periods, with 30 participants per participating center in each period. This sample size was chosen to ensure sufficient variation in user experiences for a usability study while remaining feasible within the available study duration and recruitment capacity of the participating sites. No formal power calculation was applicable as this study did not aim to detect statistical differences but to explore usability and user perceptions.

#### Quantitative Data Analysis

The Likert-scale data of the MAUQ were summarized using medians and IQRs given the skewed data distribution as visually assessed via histograms. Other categorical data were represented as relative distribution (frequencies and percentages) per item. Demographic variables were assessed for normality and reported as means with SDs, medians with IQRs, or ranges depending on distribution. No formal hypothesis testing was performed; all analyses were descriptive. Microsoft Excel and SPSS Statistics for Windows (version 28; IBM Corp) were used for all analyses.

#### Qualitative Data Analysis

Qualitative data from focus groups, in-depth interviews, and the 4 open-ended survey questions were analyzed and systematically coded using thematic analysis [27]. Participant responses were summarized as codes (ie, keywords). Codes with similar meanings were grouped into overarching topics. These topics were either facilitators of or barriers to the use of Picterus JP.

Topics (ie, barriers and facilitators) were subsequently organized into themes. These themes could follow the structure of the open-ended questions (deductive) or emerge from the data themselves (inductive). This approach allowed for a comprehensive and integrated analysis of all qualitative sources.

Coding was independently performed by country by local researchers. Additionally, one author (FT) coded all datasets of both countries to ensure consistency across contexts and maintained a manual codebook in Microsoft Excel that was iteratively refined. Another author (TdV) reviewed a sample of the coded data and confirmed agreement with the chosen topics and thematic structure.

Quantitative MAUQ results are presented first, followed by qualitative findings that provide additional context to these results.

## Results

### Participants

In total, 123 parents completed the MAUQ and open-ended questions. Table 1 shows participant characteristics. The sample included both first-time and experienced parents, with a relatively high proportion (78/123, 63.4%) of participating parents having completed higher education. In total, 16 parents were approached for in-depth interviews: 12 (75%) participated after 2 reminders across the 3 centers. Data saturation was reached after approximately 5 interviews.

**Table 1:** Participant characteristics: neonate and parent dyads.

Variable		The Netherlands (n=93)	Israel (n=30)
Hospitals, n		2	1
**Parents per period, n (%)**			
	2023	62 (66.7)	0 (0)
	2024 and 2025	31 (33.3)	30 (100)
Interviews with parents, n		6	6
Gestational age (wk), median (IQR)		39 (38-40)	39 (38-40)
Postnatal age category (h), most common category (range of reported ages)		24-48 (12-144)	24-48 (12-120)
Birth weight (g), mean (SD)		3466 (570)	3552 (339)
Female sex, n (%)		44 (47.3)	14 (46.7)
**Maternal parity,** **n (%)**			
	First	14 (15.1)	11 (36.7)
	Second	48 (51.6)	7 (23.3)
	Third or higher	31 (33.3)	12 (40)
**Parent educational level,** **n (%)**			
	Basic education	1 (1.1)	0 (0)
	Intermediate education	25 (26.9)	4 (13.3)
	Advanced education	56 (60.2)	22 (73.3)
	Unknown	11 (11.8)	4 (13.3)

The 2 focus groups included 12 nurses, all Dutch and female. Nurses had sometimes observed when parents used the app as they had been caring for mother and infant or had heard parents discuss app use afterward. Table 2 shows nurse characteristics. Overall, the nurses were relatively young and had few years of work experience.

**Table 2:** Characteristics of the registered nurses who participated in 1 of the 2 focus groups.

Variable			Focus group 1 (n=5)		Focus group 2 (n=7)
Location			Frisius Medical Center, Leeuwarden, the Netherlands		University Medical Center Groningen, the Netherlands
Female sex, n (%)			5 (100)		7 (100)
Age (y), median (IQR)			24 (24-26)		27 (23-34)
Work experience (y), median (range)			0.5 (0.3-15)		5 (0-12.5)
**Frequency of witnessing Picterus app being used by parents, n**					
	1 time	1		4	
	1-5 times	2		3	
	5-10 times	1		0	
	≥10 times	1		0	

### Quantitative Usability: MAUQ

**Figure 2:**
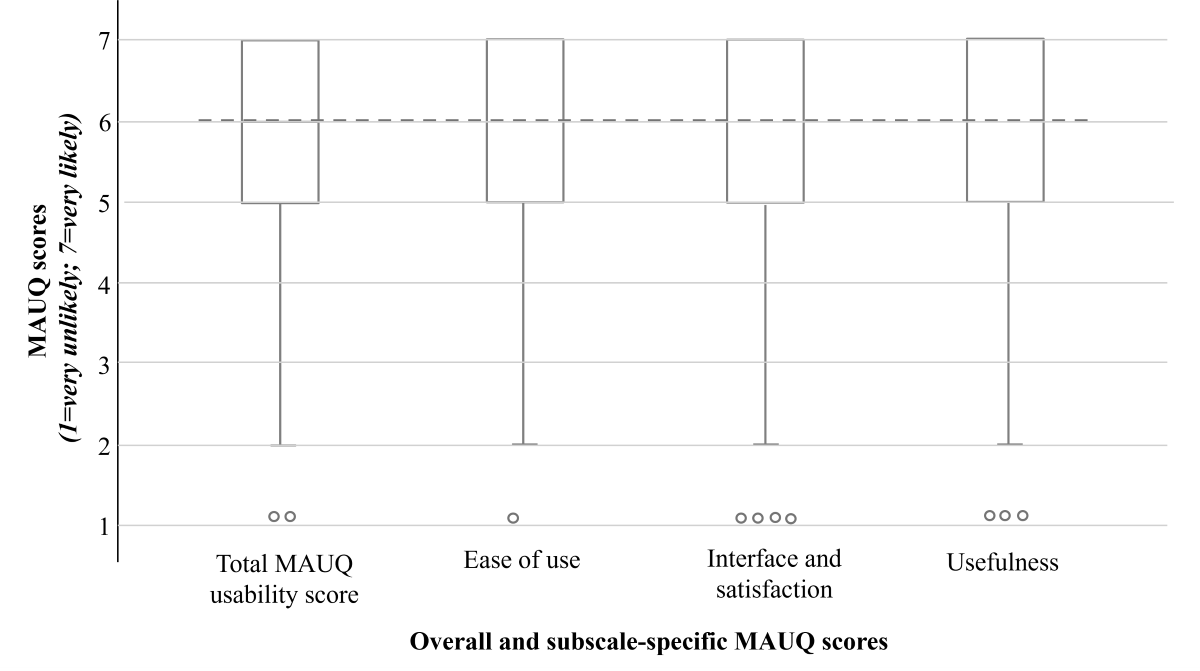
Total parental mHealth App Usability Questionnaire (MAUQ) scores and scores on the 3 subscales. The median is marked by the horizontal dotted line. The boxes are limited by the 25th and 75th percentiles. The whiskers (┴) represent the lowest and highest irradiance within 1.5 times the interquartile distance below the box. Extreme outliers (○) indicate participants who gave the lowest possible rating (ie, a score of 1) on the statements.

Figure 2 shows the median MAUQ usability score of 6 (IQR 5-7) on a scale from 1 to 7 on the MAUQ. Similarly, the median scores for the subscales of ease of use, interface and satisfaction, and usefulness were 6 (IQR 5-7). There were no differences in overall median MAUQ scores or MAUQ scores per subscale between the 2 countries, among the 3 hospitals, or between both study periods.

Parents expressed the most positive attitudes toward the statements that Picterus JP was easy to learn and improved their access to health care, with a median MAUQ score of 7 (IQR 6-7). All other statements had a median MAUQ score of 6. Table S1 in Multimedia Appendix 1 shows all 17 MAUQ statements and their corresponding median scores.

### Qualitative Usability: Thematic Analysis

#### Themes, Facilitators, and Barriers

Table 3 shows the results of the thematic analysis. Three themes were identified: “intention to use Picterus JP,” “neonatal well-being,” and “guidance and instructions.” The first 2 themes were deductive, corresponding to 2 open-ended questions, whereas the last theme was inductive and emerged from the interviews and focus groups but was also supported by the responses to the open-ended survey questions. Within these themes, 13 facilitators and 10 barriers were identified.

**Table 3:** Facilitators of and barriers to using the Picterus Jaundice Pro (JP) app: insights from parents and nurses^a^.

Theme	Facilitators	Barriers
Intention to use Picterus JP	Easy and simple to use (P^b^, I^c^, and N^d^) Home access (P and I) Feeling in control (P, I, and N) Reassuring for extra monitoring (P and I)	Difficulties obtaining measurements (P, I, and N) Doubts about relevance (I and N) Doubts about reliability (P and I) Reminders needed (I and N)
Infant well-being^e^	Quick (P and N) Comfortable (P) Safe (P) Minimally intrusive (P) Usable at own convenience (P and I)	Prolonged undress (P and I) Neonate too upset (P and I) Camera flash (P and I)
Guidance and instructions	Easy to understand (P, I, and N) Self-explanatory (P, I, and N) Quick to explain (P and N) In-person explanation (I and N)	Unclear for parents what to do when a problem occurs (P, I, and N) Uncertainty among parents regarding what to do with results (P, I, and N) In-app guidance easy to miss (P, I, and N)

^a^This table presents perceived topics divided into either facilitators of or barriers to using the Picterus JP app. Topics were grouped into 3 themes. Within themes, topics were ordered from most to least frequently mentioned.

^b^P: parents (responses from the survey; n=123).

^c^I: interviewed parents (n=12).

^d^N: nurses who participated in the focus groups (n=12).

^e^The theme “infant well-being” reflected factors related to the infant’s comfort and safety as perceived by parents but did not indicate actual clinical risk that was reported during the study.

#### Open-Ended Questionnaire Responses From Parents

Table 4 presents the 4 open-ended questions for parents. A total of 87.7% (100/114) of the parents responded affirmatively to the first question (“Would you use the app?”). For the second question, 81.7% (98/120) of the parents reported no concerns about their infants’ comfort while using the app. A common facilitator in both questions was that Picterus JP was “easy, simple, and quick to use.” Common barriers included difficulty obtaining a valid measurement and specific concerns about their baby, particularly when using the app took longer than expected. Parents worried that prolonged undressing might result in hypothermia or cause the neonate to become upset. Of the 22 parents who expressed concerns, 20 (87%) reported having encountered difficulties using the app.

**Table 4:** Open-ended questions^a^.

Question	Yes, n (%)	No, n (%)
Would you use the app? (n=114)	100 (87.7)	14 (12.3)
Concerned about neonates comfort? (n=120)	22 (18.3)	98 (81.7)
Did you encounter any difficulties using the app? (n=120)	67 (55.8)	53 (44.2)
Do you have any other comments? (n=123)	50 (40.7)	73 (59.3)

^a^Missing data were excluded from the analysis.

For the third question (“Did you encounter any difficulties using the app?”), 55.8% (67/120) of the parents reported having experienced an issue. Textbox 1 shows the reasons for difficulties, which varied between study periods and countries and were either device-related or user-related issues. Device-related difficulties referred to issues in which parents reported having received an error message. For example, when the photo quality was insufficient for analysis, Picterus JP could display a message such as “ambient light insufficient” or “difficulty focusing.”

**Parental difficulties when using the Picterus Jaundice Pro app for the first time organized from most to least frequent.**

**Device-related difficulties**

- Issue with focusing and/or ambient light
- Miscellaneous error messages (ie, freezing or calibration card not recognized)
- Unknown error
- Phone not compatible

**User-related difficulties**

- Baby factors (ie, upset or moving)
- Finding the instructions unclear
- Using the calibration card incorrectly
- Miscellaneous parental factors (ie, hand tremors)

User-related difficulties included cases in which the infant was too “uncooperative” to obtain sufficient photos or parents acknowledged incorrect use of Picterus JP (eg, displacing the calibration card).

During the first study period in the Netherlands (2023), 58.1% (36/62) of the parents primarily encountered device-related difficulties for various reasons. During the second period in the Netherlands (2024), 32.3% (10/31) of the parents reported difficulties, which were primarily user related, whereas in Israel (2025), 76.7% (23/30) of the parents reported difficulties, and these were mostly device related. In Israel, 30.4% (7/23) of these parents indicated an issue in which ambient light was insufficient. Multiple attempts were made to improve ambient light. Retrospective analysis of these photos indicated that the issues in these cases were often related to the quality of the camera and the saved file format of the specific phone model being used.

Of the 105 parents who attempted the measurement again, even though this was not required within the scope of the study, 79 (75.2%) reported that they were able to obtain a bilirubin estimate. Similarly, 70% (21/30) of the Israeli parents reported a successful measurement after further attempts.

The fourth question (“Do you have any additional comments?”) resulted in 50 responses that were integrated into the thematic analysis (Table 3). Some comments addressed misunderstandings and suggestions regarding the instructions and in-app guidance (ie, correct use of the calibration card), whereas others expressed uncertainty about interpreting the results. Additionally, satisfaction with Picterus JP was expressed. All emerging topics were closely aligned with findings from the other survey questions, interviews, and focus groups.

#### Findings From Focus Groups With Nurses and Interviews With Parents

Because themes and topics overlapped, results from parents and nurses are presented together. The identified topics in focus groups with nurses and in-depth interviews with parents supported and further contextualized the topics previously reported by parents in the questionnaires. This led to the identification of 3 additional topics (Table 3).

An additional identified facilitator was the perceived value of receiving an in-person explanation or demonstration of the app. There were 2 additional barriers. First, parents needed reminders to use the Picterus JP app. Another issue was that in-app guidance could easily be missed, which delayed or disrupted the process. All 3 of these topics were mentioned by both nurses and parents. Table S1 in Multimedia Appendix 3 presents supporting quotes from nurses.

#### Theme 1: Intention to Use Picterus JP

Consistent with questionnaire findings, ease of use was frequently mentioned as a facilitator by parents and nurses (quote 1: Multimedia Appendix 3). However, more unique to this part of the data collection, both nurses and parents noted that reminders to use Picterus JP were often necessary (quote 2).

Parents appreciated the ability to monitor their neonate for jaundice themselves but questioned its added relevance alongside regular health care visits. Parents stated that it had to be very clear for them what they had to do with the results. In the interviews, they emphasized the need for clear guidance. Nurses acknowledged the relevance as well, noting that their clinical experience and recognition of early signs of jaundice served as important reminders for parents (quote 3).

#### Theme 2: Neonatal Well-Being

Similarly to the answers from parents, nurses mentioned that taking a photo using Picterus JP was perceived as quick and nondistressing for the neonate. Similarly to the questionnaire, parents expressed a preference for additional warnings about the camera flash to remind them to cover their neonates’ eyes (quote 4). Nurses expressed no concerns about infant well-being during the use of Picterus JP when asked about disadvantages or improvements that could be made.

#### Theme 3: User Guidance and Instructions

User guidance and instructions were most prominently discussed during interviews and focus groups. Oral explanations were preferred over written instructions even though, in general, the latter were considered clear (quote 5). Nurses found the instructions easy to explain, and often parents agreed that understanding what to do was straightforward (quote 6).

Interestingly, as was often reported in the survey and also shown in Textbox 1, when parents were unsuccessful in obtaining a measurement, it was not always clear to them what had gone wrong (quote 7). Some parents admitted in hindsight that they had not fully paid attention to the in-app guidance or that they had misunderstood the instructions (quote 8). Some nurses reported that, at times, they were unsure why a measurement had failed. In contrast, other nurses stated that the cause of failure was often clear to them as observers, whereas parents appeared to have overlooked the in-app guidance (quote 9).

Overall, nurses in the focus groups were positive about the potential parental use of Picterus JP in the home setting (quote 10).

## Discussion

### Principal Results

We determined how parents perceived the usability and user-friendliness of Picterus JP, a novel smartphone app to allow for early detection of imminent SNH. Our results show that parents rated Picterus JP highly on usability, supported by a median MAUQ score of 6 (IQR 5-7) out of 7. Parents described Picterus JP as easy, simple, and quick to use.

A considerable number of parents reported either technical difficulties or movement of their baby as reasons for failing to obtain a result on the first use of Picterus JP.

Technical difficulties reported by users may be partly explained by the variation in smartphone devices used across study sites despite the fact that these devices passed Picterus JP’s compatibility check. However, some phones (eg, budget models) may still be more sensitive to ambient lighting conditions. In Israel, all parents used the same midrange phone provided by the study team, whereas in the Netherlands, varied personal devices were used. This difference may explain the lower incidence of technical difficulties in the Netherlands than in Israel. Furthermore, within the Dutch sites, technical difficulties decreased in the second study period, possibly due to ongoing software improvements of Picterus JP.

In addition to technical difficulties, some parents (22/120, 18.3%) expressed concerns regarding neonatal well-being during app use. Frequent topics were concerns about the infant becoming cold or upset. Most of these parents (20/23, 87%) reported having encountered difficulty using Picterus JP on their first attempt. This may suggest a possible connection between prolonged attempts and parental concerns, but this was not formally studied.

Although parents and nurses found the Picterus JP instructions clear, they reported that instructions could be overlooked or misunderstood. It is possible that, in some cases, unsuccessful attempts were related to skipped or misunderstood instructions rather than technical failure alone. Picterus JP offers multiple instructions: symbols on calibration cards and the app launch screen, online text, and in-app pop-ups. It is tempting to speculate that passive instructional methods may not fully support parents in busy postpartum settings.

Nonetheless, most parents (100/114, 87.7%), even among those who encountered technical difficulties or had concerns, stated that they would use the app at home. This implies that, despite initial barriers, overall acceptability and perceived usefulness of Picterus JP in a home setting are high, which are both essential prerequisites for its implementation. Given the near-universal smartphone use in our study population, eventual barriers to implementation are unlikely to stem from limited access to or lack of experience with smartphones [28].

Notably, the nurses participating in focus groups were young and had limited work experience, reflecting the composition of junior and senior staff typically present during shifts. Demographic characteristics, with younger nurses potentially being more technologically savvy, may influence generalizability but also improve comparability with similarly aged parents.

Nevertheless, all nurses had experience with SNH screening and regarded Picterus JP as relevant and useful. They emphasized that parents engaged more with Picterus JP when its relevance was clear to them too. Moreover, insights from nurses helped contextualize parental responses as they frequently observed parents during the use of Picterus JP. This underlines the importance of incorporating multiple perspectives in usability studies.

### Comparison With Prior Work

While many SNH screening applications have been developed over the years, our findings align with broader digital health literature showing that the main challenges of home-based screening tools lie in usability [29]. Despite the widely recognized need for accessible screening tools to detect SNH, still few clinically validated alternatives currently exist. Although noninvasive screening can be performed using TcB devices, these devices are rather costly and currently not available in home settings [7]. To save costs, 2-color and multicolor icterometers (ie, the BiliStrip and Bili-Ruler, respectively) have been developed with rather acceptable screening performances and interrater reliability [14,17]. In parallel with these icterometers, other smartphone apps to optically estimate hyperbilirubinemia have been developed and studied over the past years [7,15,16]. Except for Picterus JP, to our knowledge, none have currently published usability results or reached a clinical implementation phase and obtained a CE mark or Food and Drug Administration certification.

Our findings on the usability of Picterus JP are consistent with those of the 2 prior studies to date in which the use of Picterus JP was assessed by HCPs. HCPs perceived Picterus JP as an easy-to-use, useful, and time-saving tool in the management of neonatal jaundice [30,31]. Our study builds on previous work by including the perspectives of both HCPs and parents in high-income countries.

Usability studies are a relevant step for implementation given that apps that are considered easy to use have yielded higher acceptability and satisfaction ratings [32]. However, studies linking app usability to clinical outcomes in pediatrics remain scarce and are mainly focused on chronic disease management [33,34]. The MAUQ is a validated and reliable instrument specifically developed to evaluate mHealth apps used by patients and HCPs, unlike more general instruments such as the System Usability Scale and the Post-Study System Usability Questionnaire [26].

The MAUQ has increasingly been adopted in recent years and used for studies across diverse health care settings worldwide [35]. High MAUQ scores have been associated with proper test performance in another mHealth screening app [36] This suggests that high usability scores as measured using the MAUQ could be a relevant indicator for evaluating the feasibility of successful implementation of a screening mHealth tool such as Picterus JP.

### Limitations

This study has several limitations. First, we used convenience sampling, which may limit representativeness, although saturation was observed in the open-ended responses, interviews, and focus groups. The design may have led to selection bias: participants were motivated to take part, comfortable with smartphone use, less frequently first-time parents, and noticeably highly educated on average. We did not observe a clear association between educational level and MAUQ score or between educational level and parental experience (ie, failure to obtain a measurement). This does not eliminate the concern that less technologically savvy or lower-literacy parents may have been systematically underrepresented. Future studies should, therefore, specifically evaluate usability and feasibility in more diverse populations and care settings, including lower-income countries.

Second, the observational nature of our study design limited us to reporting descriptive findings rather than statistical cross-site comparisons or adjusting for potential confounders.

Third, MAUQ scores were generally high (median 6, IQR 5-7 on a 7-point scale) but with no complete concentration at the maximum score, suggesting no clear evidence of a ceiling effect.

Finally, the app was typically used for only 1 measurement in a hospital setting, with limited instruction with the purpose of generalizing the outcomes. One in-hospital measurement with either the study team or an HCP nearby may introduce performance bias and limit ecological validity. Although the study team was not always directly present during measurements, the potential influence of supervision was not systematically assessed. It is important to note that, in real-world settings, parents are also likely to use the app in interaction with HCPs such as maternity care assistants, who provide daily postpartum care in the Netherlands, or during telehealth consultations. The intended repeated daily use of Picterus JP in variable settings might influence user experience differently. Although repeated use might expose the user to other user- and device-related difficulties, familiarity through guidance and practice may also enhance perceived parental usability of the app.

### Strengths

To the best of our knowledge, this is the first study to assess the usability of a smartphone-based bilirubin screening tool from the perspective of both parents and HCPs. Furthermore, the multicenter and multicultural setting spanning 2 countries and 3 hospitals adds to the potential generalizability of the results to different health and care systems as similar usability patterns emerged across diverse user groups. In addition, we used a validated instrument (MAUQ) to evaluate usability, which enhances the reliability and comparability of our findings.

### Conclusions

Our findings show that parents and nurses highly appreciated the Picterus JP app despite some device- and user-related difficulties. Our findings highlight the importance of ongoing technological improvements, clear user guidance, and proactive support to improve usability and user experience to successfully facilitate the implementation of parental use of Picterus JP in neonatal jaundice management.

If future studies confirm the clinical accuracy and reliability of parental use of Picterus JP in different populations and health and care systems, daily noninvasive home screening could become feasible. As such, the use of Picterus JP may enable earlier detection of imminent SNH, allowing for timely intervention for at-risk neonates, reducing the emotional and logistical burden on families, and supporting clinical decision-making by HCPs.

## Data Availability

The datasets generated and analyzed during this study are not publicly available due to participant privacy and ethical restrictions but are available from the corresponding author on reasonable request. Data can be shared in fully anonymized form for noncommercial research purposes.

## Appendix

Multimedia Appendix 1 Statements and results of the mHealth App Usability Questionnaire.

Multimedia Appendix 2 The topic list used for interviews and focus groups.

Multimedia Appendix 3 Quotes from interviews and focus groups.

## Abbreviations

HCP: health care professional
JP: Jaundice Pro
MAUQ: mHealth App Usability Questionnaire
mHealth: mobile health
SNH: severe neonatal hyperbilirubinemia
TcB: transcutaneous bilirubin
TSB: total serum bilirubin
UMCG: University Medical Center Groningen
