# Supplementary material for: Evaluating the Picterus Jaundice Pro App for Neonatal Jaundice Screening by Parents: Prospective Mixed Methods Study
Source: JMIR Pediatr Parent. 2026 Jul 27;9:e83077. doi: 10.2196/83077 (PMC13458348; doi:10.2196/83077)
Supplement: Supplementary file 1 [file pediatrics_v9i1e83077_app1.docx]

**Table S1. Statements and results of the mHealth Usability Questionnaire (MAUQ)**

|  | **MAUQ subcategories & statements^a^** | Median | IQR |
| --- | --- | --- | --- |
|  | **1. Ease of Use** | 6 | [5-7] |
| 1. | The app was easy to use. | 6 | [5-7] |
| 2. | It was easy for me to learn to use the app. | 7 | [6-7] |
| 3. | The navigation was consistent when moving between screens. | 6 | [6-7] |
| 4. | The interface of the app allowed me to use all the functions (such as entering information, responding to reminders, viewing information) offered by the app. | 6 | [5-7] |
| 5. | Whenever I made a mistake using the app, I could recover easily and quickly. | 6 | [4-7] |
| 6. | I like the interface of the app. | 6 | [5-7] |
|  | **2. Interface & satisfaction** | 6 | [5-7] |
| 7. | The information in the app was well organized, so I could easily find the information I needed. | 6 | [5-7] |
| 8. | The app adequately acknowledged and provided information to let me know the progress of my action. | 6 | [5-7] |
| 9. | I feel comfortable using this app on my child. | 6 | [5-7] |
| 10. | The amount of time involved in using this app has been fitting for me. | 6 | [5-7] |
| 11. | I would use this app again. | 6 | [5-7] |
| 12. | Overall, I am satisfied with this app. | 6 | [5-7] |
|  | **3. Usefulness** | 6 | [5-7] |
| 13. | The app would be useful for the health and well-being of my child. | 6 | [6-7] |
| 14. | The app improved my access to healthcare services when my child is jaundiced. | 7 | [6-7] |
| 15. | The app helped me manage the health of my child effectively. | 6 | [4-7] |
| 16. | This app has all the functions and capabilities I expected it to have. | 6 | [4-7] |
| 17 | ~~I could use the app even when the Internet connection was poor or not available.~~^b^ | NA | |
| 18. | This mHealth app provides an acceptable way to receive healthcare services, such as accessing educational materials, and performing an assessment on my child. | 6 | [4-7] |
| ^a^Scored by parents from 1—strongly disagree; 2—disagree; 3—somewhat disagree; 4—neither agree nor disagree; 5—somewhat agree; 6—agree; 7—strongly agree  ^b^Statement 17 was deleted during analysis as we already know Picterus JP does not work without internet connection and did not study offline use. | | | |
