# Supplementary material for: Evaluating the Picterus Jaundice Pro App for Neonatal Jaundice Screening by Parents: Prospective Mixed Methods Study
Source: JMIR Pediatr Parent. 2026 Jul 27;9:e83077. doi: 10.2196/83077 (PMC13458348; doi:10.2196/83077)
Supplement: Supplementary file 2 [file pediatrics_v9i1e83077_app2.docx]

**Topic list Parental User Experience study**

**Instructions**

- Ensure all themes (e.g., general experience, instructions) are covered during the discussion. The listed questions are meant to guide the conversation, not to be followed rigidly.
- Please record the conversations, transcribe them, and then translate them into English if possible.
- Begin the interview or focus group with an introduction and a consent round. For example: "My name is [name], my profession is [profession], my use of the app was [description], and I consent to this interview."
- Conclude by asking if the participant would like to share anything that hasn’t been covered.

**Parents (5-15 minutes)**

**General Experience**

- What benefits did you experience with the app?
- What disadvantages did you experience with the app?

**Instructions**

- How did you find the instructions/what instructions did you receive?
  - Did you use the instruction screen within the app?
- What information did you find useful?
- What type of information did you receive? (verbal, non-verbal like a leaflet, from the researcher, icons, or via the app itself?)
- Did you feel you received enough information?
- Looking back, what information would you have liked to receive more of?

**Reliability and issues**

- Did the app ever not work?
  - Did you use the app more than once?
- Was it clear what you needed to do if the app didn’t work?
- What was needed in those situations?

**Future**

- What would you tell parents of newborns about using a jaundice app?
- Would you recommend using the app at home?
- Are there things you think could be improved?
- Is there anything else you’d like to share or ask?

**Focus group with health care providers (15-20 minutes)**

**General Experience**

- How did you interact with the app? (Did you see it being used, use it yourself, or hear parents talk about it?)
- Did you have positive or negative experiences?

**Instructions**

- Did you know how the app worked yourself?
- What information did you find useful? (Explanation from a researcher/colleague, the app itself, written information)
- Did you feel the instructions for parents were clear?
- What information do you think parents needed?

**Reliable Use**

- How did parents handle using the app? (Was it easy/difficult? Did they use it correctly?)
- Did you feel the app was used correctly by parents?
- Did the app ever not work?
- Was it clear to parents what to do if the app didn’t work?
- Did you ever help with this?
- What was needed to get the app working when it didn’t work the first time?

**Future**

- What would you tell healthcare professionals (maternity nurses, midwives) who visit parents of newborns using a jaundice app?
- Would you recommend using the app at home?
- Are there things you think could be improved?
- Is there anything else you’d like to share or ask?
