# Supplementary material for: Evaluating the Picterus Jaundice Pro App for Neonatal Jaundice Screening by Parents: Prospective Mixed Methods Study
Source: JMIR Pediatr Parent. 2026 Jul 27;9:e83077. doi: 10.2196/83077 (PMC13458348; doi:10.2196/83077)
Supplement: Supplementary file 3 [file pediatrics_v9i1e83077_app3.docx]

| **Table S2: Quotes from interviews and focus groups** | |
| --- | --- |
| Quote 1 – Parent, during in-depth interview | *Quick, easy. You just have to download it and then you could scan right away. That is quite handy.* |
| Quote 2 – Nurse, during focus group | *Only, I did notice, we work with postpartum women, and of course, they are focused on completely different things. So when I said, ‘Shall we quickly do it during a feeding moment?’ it was like, ‘Oh yeah, oh yeah.’ You had to remind them more often to make sure they actually used it.* |
| Quote 3 – Nurse, during focus group | *What I also noticed: people found the app and participating more interesting when they noticed that it was useful for their child. But if it didn’t fit their medical condition or hospital stay at all, they often didn’t pay attention to it anymore.* |
| Quote 4 – Parent, during in-depth interview | *One area for improvement is that it should indicate when a flash is about to happen, because suddenly there’s a flash right in the baby’s face. It would be helpful to have a warning, like asking you to close the baby’s eyes before the flash.* |
| Quote 5 – Nurse, during focus group | *One patient may have better reading comprehension than another, and you have to deal with all cases here, educational levels and so on.* |
| Quote 6 – Parent, during in-depth interview | *Well, it just said to download the app, so we did. Then it said to scan the card, that was it. Nice and simple, just two steps.* |
| Quote 7 – Parent, during in-depth interview | *No, I don't really remember what the problem even was. We just tried again and again until it worked.* |
| Quote 8 – Parent, during in-depth interview | *I thought, oh, I have to set up the camera first with that (i.e., calibration) card, it has to scan that card first (i.e., without the infant).* |
| Quote 9 – Nurse during focus group | *…but I did notice that parents didn’t realize ”oh, I need to pay attention to this”. […] I would already see: you’ve been holding the angle (i.e., of the phone camera) wrong the whole time.* |
| Quote 10 – Nurses, during focus group | *N1: […]it does have an addition. I really see the benefit of it. So long it is worth the effort (i.e., for parents). N2: I’m just too curious myself, so I would definitely want to use this at home.* |
